# Hyperspectral Analysis of Soil Total Nitrogen in Subsided Land Using the Local Correlation Maximization-Complementary Superiority (LCMCS) Method

**DOI:** 10.3390/s150817990

**Published:** 2015-07-23

**Authors:** Lixin Lin, Yunjia Wang, Jiyao Teng, Xiuxiu Xi

**Affiliations:** 1 School of Environment Science and Spatial Informatics, China University of Mining and Technology, Xuzhou 221116, China; E-Mails: rsande@163.com (L.L.); zgkdcumt8@163.com (J.T.); xxxxiuxiu@163.com (X.X.); 2 National Administration of Surveying, Mapping and Geo-Information (NASG), Key Laboratory of Land Environment and Disaster Monitoring, Xuzhou 221116, China

**Keywords:** hyperspectral reflectance, ASD FieldSpec spectroradiometers, local correlation maximization-complementary superiority, soil total nitrogen, subsided land

## Abstract

The measurement of soil total nitrogen (TN) by hyperspectral remote sensing provides an important tool for soil restoration programs in areas with subsided land caused by the extraction of natural resources. This study used the local correlation maximization-complementary superiority method (LCMCS) to establish TN prediction models by considering the relationship between spectral reflectance (measured by an ASD FieldSpec 3 spectroradiometer) and TN based on spectral reflectance curves of soil samples collected from subsided land which is determined by synthetic aperture radar interferometry (InSAR) technology. Based on the 1655 selected effective bands of the optimal spectrum (OSP) of the first derivate differential of reciprocal logarithm ([log{1/R}]′), (correlation coefficients, *p <* 0.01), the optimal model of LCMCS method was obtained to determine the final model, which produced lower prediction errors (root mean square error of validation [RMSEV] = 0.89, mean relative error of validation [MREV] = 5.93%) when compared with models built by the local correlation maximization (LCM), complementary superiority (CS) and partial least squares regression (PLS) methods. The predictive effect of LCMCS model was optional in Cangzhou, Renqiu and Fengfeng District. Results indicate that the LCMCS method has great potential to monitor TN in subsided lands caused by the extraction of natural resources including groundwater, oil and coal.

## 1. Introduction

In recent years, land subsidence caused by the extraction of natural resources such as groundwater [1,2], oil [3] and coal [4,5] has created severe and widespread hazards in China, resulting in new ecological and environmental issues such as soil degradation and loss of biodiversity. Nitrogen is necessary for all known forms of life on Earth, being present in the environment in a wide variety of chemical forms including organic nitrogen, ammonium, nitrite and nitrate. Organic nitrogen may be in the form of a living organism, humus or the intermediate products of organic matter decomposition. The nitrogen cycle processes transform nitrogen from one form to another [6,7], therefore monitoring of TN plays an important role in soil restoration programs, which has stirred the interest of many scholars and recently resulted in a series of achievements [8,9]. However, most successful approaches are based on traditional chemical testing methods, which tend to be time consuming, laborious, and expensive [10]. Consequently, researchers have sought real-time methods for monitoring of TN content of soils.

Hyperspectral remote sensing provides an abundance of spectral information, which suggests a potential method for estimating soil properties [11,12,13,14,15]. Compared with traditional laboratory methods, hyperspectral techniques are more rapid and less costly, and can eliminate the need for sample preparation and chemical reagents [11,16]. The TN content can significantly affects the shape and nature of a soil spectral reflectance spectrum. The wide spectral range suitable for estimating TN content suggests that TN is an important soil constituent across the entire spectrum [17,18]. Therefore, many studies have reported on various TN monitoring models based on hyperspectral remote sensing [19,20]. For example, Dalal *et al.* [19] and Morra *et al.* [20] both used stepwise multiple linear regression for the rapid quantification of TN contents. Sun *et al.* [21] estimated TN using wavelet analysis and transformation. Zheng *et al.* [22] quantified TN content through near-infrared reflectance (NIR) spectroscopy and use of a back-propagation (BP) neural network.

Using modern sensors, significant studies have been carried out on spectral characteristics of water, plants and soils, forming a scientific basis for the application of hyperspectral remote sensing technology in subsided land soils [7,23,24]. Some major achievements were analyzed briefly (see Table 1).

Partial least squares regression (PLS regression) has the advantages of treating very large data matrices such as those typically employed with hyperspectral reflectance data; therefore, this technique has been successfully applied to spectral data for predicting soil nitrate [25] and organic matter content [26,27], and also has been employed for predicting TN [28,29]. Shi *et al.* [30] compared three methods for estimating TN content with visible/near-infrared reflectance (Vis/NIR) of selected coarse and heterogeneous soils, and the PLS regression model performed best. Chang *et al.* [31] integrated near-infrared reflectance spectroscopy (NIRS) and used PLS regression to predict several soil properties including TN. In general, many studies have confirmed that PLS regression was one of the most efficient methods used for constructing reliable models in a wide range, including hyperspectral remote sensing [32].

**Table 1:** Major research works on water, plants and soils using modern sensors.

Research Field	Sensors	Factor Monitored	Application	Reference
Water	Ocean Optics USB4000	Chlorophyll a	Estimation of chlorophyll-a in turbid inland waters	[33]
	ASD	Fucoxanthin, zeaxanthin, chlorophyll a and chlorophyll b	Quantification of diatom biomass in Microphytobenthic (MPB) biofilms (non-destructively)	[34]
	ASD, ATM-2	Grain size	Characterization and management of the beach environment	[35]
Plants	Airborne HyMap	Foliar nitrogen	prediction of sagebrush canopy nitrogen from an airborne platform	[36]
	Perkin Elmer Lamdba 19	Leaf pigment, Chlorophyll, Carotenoid, Nitrogen, Carbon	Spectroscopy of plant biochemistry	[37]
	ASD	Leaf chlorophyll	Retrieval of spatially-continuous leaf chlorophyll content	[38]
	ASD	Major plant species	Classification of Hyperspectral images	[39]
	ASD	Fusarium circinatum Stress	Early detection of Fusarium circinatum-induced stress in Pinus radiata seedlings.	[40]
	ProSpecTIR-VS, ASD	Plant stress	The Plant Stress Detection Index (PSDI) used as plant stress indicator	[41]
	ASD	Mangrove leaves	Mangrove classification	[42]
	ASD	Water stress	Prediction of Grain and biomass yield of wheat based on water stress indices	[43]
	ASD, Ocean Optics (QE65000, Jaz)	pH	Determination of pH in Sala mango	[44]
	ASD	Zn content	Monitoring Zn nutrient levels under field conditions	[45]
	ASD	Leaf chlorophyll	Validation of satellites’ vegetation products	[46]
Soils	ASD	Soil nitrogen, carbon, carbonate, and organic matter	Assessing nitrogen, carbon, carbonate and organic matter for upper soil horizons (non-destructively).	[6]
	ALPHA FT-IR	Soil carbon	Soil carbon validation at large scale	[13]
	HySpex VNIR-1600	Soil carbon, nitrogen, aluminum, iron and manganese	Improvement of soil classification, assessment of elemental budgets and balances and understanding of soil forming processes and mechanisms.	[14]
	ASD	Soil bulk density, moisture content, clay, silt, and sand	Estimating the physical properties of paddy soil	[47]

Adaptive neuro-fuzzy inference systems (ANFIS), which combine the aspects of a fuzzy system with those of a neural network, have been widely used in many fields because of its usefulness with complex nonlinear problems [48,49,50,51,52,53,54]. ANFIS has also been applied to the hyperspectral assessment of soil properties [55]. Although it is difficult to make full use of hyperspectral data because of the restriction on the number of input variables, ANFIS may be a promising technique in the field of hyperspectral remote sensing.

Although accumulated research achievements in estimating TN using hyperspectral remote sensing technology have been seen, few studies have been undertaken in areas of subsided land, which have geo-spatial, social, and environmental factors that are widespread, comprehensive, dynamic, and complicated [56,57]. In addition, almost no analysis of TN in subsided land caused by the extraction of various resources currently exists. To bridge this gap, several issues need to be considered to provide satisfactory prediction accuracy: Whether the existing TN estimation models are suitable for soils influenced by land subsidence? Noise reduction must be considered in developing hyperspectral estimation models [58,59], but how to reduce noise while retaining as much useful information as possible in remotely sensed hyperspectral data? How to realize the complementary superiority of PLS regression and ANFIS to further improve the accuracy of TN estimates?

In view of the above issues, the objective of this study was to develop a suitable method for estimating the soil TN in subsided lands. In order to achieve this goal, Local Correlation Maximization-Complementary Superiority (LCMCS) method was investigated. LCMCS takes advantages of both PLS regression and ANFIS, and can maximize the use of TN response information and eliminate the interference of noisy data. The performance of LCMCS model was compared and evaluated by the local correlation maximization (LCM), complementary superiority (CS) and PLS regression methods.

## 2. Materials and Methods

**Figure 1:**
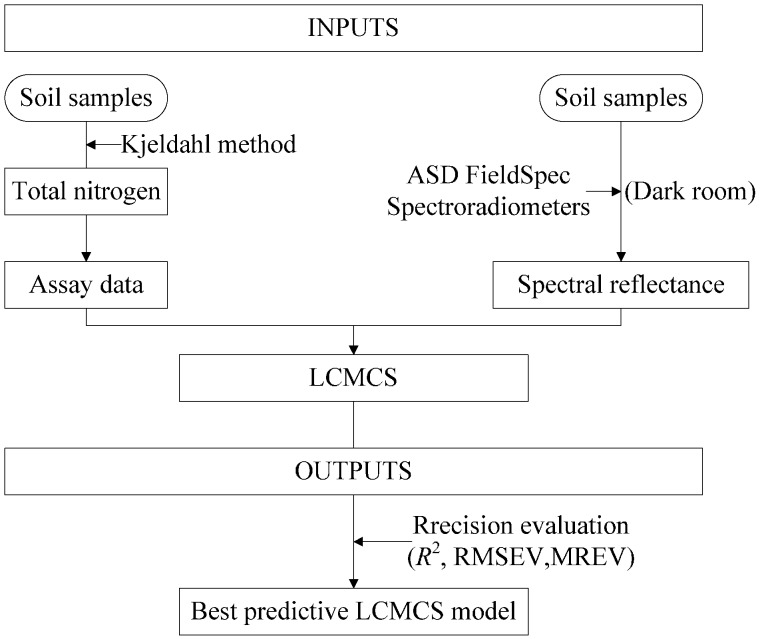
Schema showing an overview of the inputs and analysis steps of the work reported in this paper to produce the LCMCS prediction models.

The overall approach applied to the model development is shown in Figure 1. This outlines the collection of soil samples and the spectral analysis and LCMCS modelling approach.

### 2.1. Experiment

#### 2.1.1. Sample Preparation

**Figure 2:**
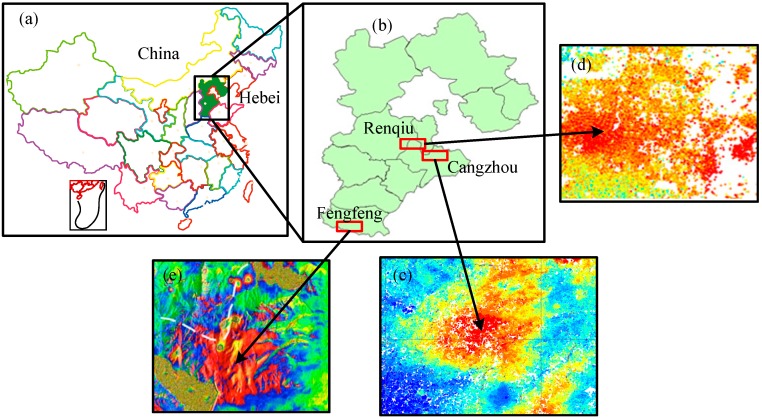
(**a**) Vicinity map of Hebei Province, China; (**b**) Vicinity map of the Changzhou, Fengfeng, and Renqiu study sites within Hebei; Soil sample collection sites from subsided land (red regions) of Changzhou (**c**); Renqiu (**d**) and Fengfeng (**e**).

The topsoil samples (0–30 cm) analyzed in this study had been randomly collected from different soil types (Table 2) at 280 randomly selected sites in the fields that had been subsided (red regions in Figure 2) of Cangzhou (Figure 2c; 38°32′ N, 116°45′ E), Renqiu (Figure 2d; 38°42′ N, 116°7′ E) and Fengfeng District (Figure 2e; 36°20′ N, 114°14′ E), all in Hebei Province, China. Subsidence had been caused by the excessive extraction of groundwater, oil and coal in these three areas, respectively. Interferometric synthetic aperture radar (InSAR) is an operational remote sensing technique to measure ground deformation with subcentimetric precision from space [60,61]. In this study, the subsidence deformation data of Cangzhou and Renqiu were obtained by permanently scattered interferometric synthetic aperture radar technology [62], while data for Fengfeng District were captured by differential synthetic aperture radar interferometry technology [63]. All 280 soil samples were air dried, gently crushed, passed through a 2 mm sieve, and then pulverized by grinding. The samples were split into two parts used for chemical analysis and spectral measurement. The percentage of TN in each soil sample was determined by the Institute of Soil Science, Chinese Academy of Sciences, Nanjing, China (measured by Kjeldahl method).

**Table 2:** Soil types in subsided land of Changzhou, Renqiu and Fengfeng.

City	Soil Types
Changzhou	Fluvo-aquic soil, Salinized fluvo-aquic soil
Renqiu	Fluvo-aquic soil, Salinized fluvo-aquic soil
Fengfeng	Cinnamon soil

#### 2.1.2. Measurement and Data Processing

An ASD FieldSpec 3 spectroradiometer (Analytical Spectral Devices, Boulder, CO, USA) was used to measure the spectra of soil samples over wavelength ranges of 350–1000 nm and 1000–2500 nm, with increments of 1.4 nm and 2 nm, respectively. The spectral resolution at 700 nm was 3 nm, and at 1400 nm and 2100 nm was 10 nm. Each soil sample was placed in a 10 cm diameter, 2 cm deep container and illuminated from above using a halogen lamp. After adjusting the zenith angle (approximately 30°) and the distance (approximately 30 cm) between the light source and soil surface, 10 scans for each sample were acquired. And white panel measurements were used as calibration. All these operations were performed in a dark room to avoid the effects of stray light [64]. By dividing the mean radiance of 10 consecutive scans by the radiance over the Spectralon panel, the spectral reflectance of the soil samples was calculated, which was regarded as the original spectrum [65].

#### 2.1.3. Spectral Transformations

Derivative processing helps reduce the influence of low-frequency noise [66,67]. In the reciprocal logarithm mode, spectra differences of the visible-light region can be highlighted and the influence of changes in illumination can be minimized [68]. In this study, each original spectral reflectance (REF) was transformed into the first derivative differential (FDR), reciprocal logarithm (log[1/R]) and the first derivative differential of reciprocal logarithm ([log{1/R}]′).

#### 2.1.4. Retrieval Model

As many studies have confirmed that PLS regression is one of the most efficient methods used in constructing reliable models in the field of hyperspectral remote sensing; therefore, this paper used PLS regression analysis to analyze the first issue of whether the existing TN estimation models are suitable for soils influenced by land subsidence. The LCM and CS methods were specifically aimed at second and third issues considered in this study. Finally, in order to solve all three issues, the LCMCS method was used to retrieve the TN content. The results were compared and evaluated.

### 2.2. Methods

#### 2.2.1. Local Correlation Maximization De-Noising Method (LCM)

**Figure 3:**
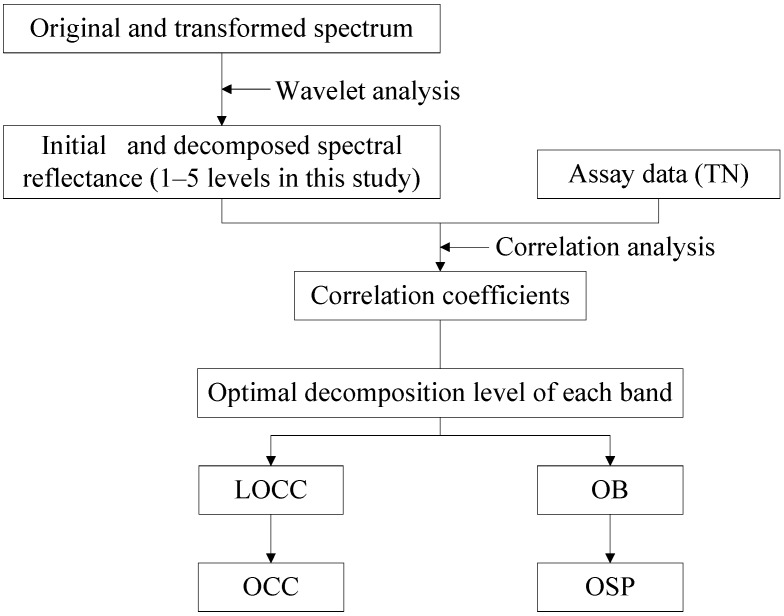
Schema showing an overview about obtaining of the optimal correlative curve (OCC) and the optimal spectra (OSP).

The soil spectral reflectance curves always have obvious burrs, which show that a large number of noisy data exist within the spectrum. This noise is also present in the transformed spectrum. How can noise be reduced while retaining as much useful information as possible? Based on the concept of local optimization, this study employed the LCM de-noising method to solve this difficult problem. The main steps of LCM are as follows:

(1) Decomposing the original and transformed spectrum into five layers using a wavelet de-noising method that is based on the Sym8 matrix function.
(2) Calculating the correlation coefficients for the measured TN content compared with both initial (including original and transformed spectrum, the same hereafter) and decomposed spectral reflectance (1–5 levels in this study), in the range of 350–2500 nm.
(3) Finding the optimal decomposition level of each band, which has the maximum correlation coefficient among initial and decomposed spectra (1–5 levels) at each wavelength; then, the corresponding correlation coefficient and decomposed band are taken as the local optimal correlation coefficient (LOCC) and optimal band (OB). After all the LOCCs and OBs are acquired, the overall LOCC and OB are used to determine the optimal correlative curve (OCC) and the optimal spectra (OSP), respectively. Finally, the OSP and OCC of original and transformed spectra are obtained, Figure 3 shows the overall approach.

#### 2.2.2. Partial Least Square Regression (PLS Regression) Method

The PLS regression method proposed by Gerlach *et al.* [69] is a mainstream, linear multiple regression method that compresses spectral data by reducing the measured collinear spectral variables to a few non-correlated latent variables or factors [70,71,72]. PLS regression algorithms have been used largely in soil analyses [13,26,27,28,29]. The basic aim of PLS regression is to build a linear model about *X* (mean-centered matrix of predictor variables; the spectral bands in this study) and *Y* (mean-centered matrix containing the response variables; the TN contents in this study). The PLS regression was carried out using the SPSS software in this study, and the number of latent variables were determined according to the prediction error in calibration [73,74]. The main principle is as follows [75]:

First, *X* and *Y* are decomposed into feature vectors in the forms of Equations (1) and (2):

(1) Y=UQ+F

(2) X=TP+E

where *U* and *T* are the score matrices, *Q* and *P* are the loading matrices, and *F* and *E* are the error matrices [76].

According to the correlation between feature vectors, a regression model is established by decomposing *X* and *Y*:

(3) U=TB+Ed

where *Ed* is the random error matrix, and *B* is the regression coefficient matrix.

Thus, if spectral vector *x* is known, the predicted TN content *y* can be obtained:

(4) $$y=x{\left(UY\right)}^{\prime }BQ$$

#### 2.2.3. Adaptive Neuro-Fuzzy Inference System (ANFIS)

**Figure 4:**
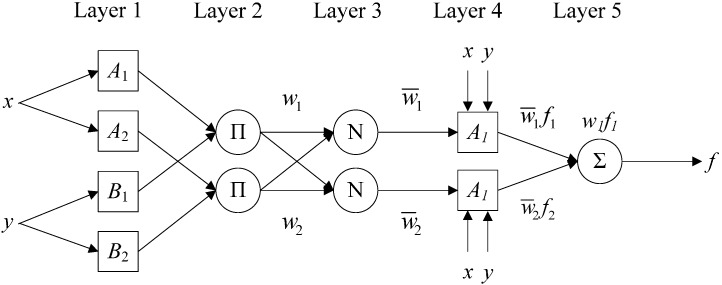
Architecture of adaptive neuro-fuzzy inference system (ANFIS).

ANFIS is an adaptive neuro-fuzzy inference machine combination of fuzzy theory with neural nets [77]. As one of the popular learning methods in neuro-fuzzy systems, a fuzzy inference system uses hybrid learning algorithms to identify the fuzzy system parameters and to train the model [78]. Figure 4 shows the ANFIS architecture with two inputs and one output, which has five layers and two rules.

Two fuzzy if-then rules [79] are given as follows:

(5) $$\text{Rule}\;1:\;\text{If}\;(x\;\text{is}\;A_{1})\;\text{and}\;(y\;\text{is}\;B_{1}),\;\text{then}\;(f_{1}=p_{1}x+q_{1}y+r_{1})$$

(6) $$\text{Rule}\;2:\;\text{If}\;(x\;\text{is}\;A_{2})\;\text{and}\;(y\;\text{is}\;B_{2}),\;\text{then}\;(f_{2}=p_{2}x+q_{2}y+r_{2})$$

Layer 1: Every adaptive node in this layer is a square node with the following node functions:

(7) $$O_{1,i}=\mu_{A_{i}}\left(x\right),i=1,2$$

(8) $$O_{1,i}=\mu_{B_{i-2}}\left(y\right),i=3,4$$

where *O*_1,1_ and *O*_1,2_ are used to grade the memberships of fuzzy sets *A* and *B*. Usually, a bell function is used as follows:

(9) $$\mu_{A_{i}}\left(x\right)=\frac{1}{1+{\left[{\left(\frac{x-c_{i}}{a_{i}}\right)}^{2}\right]}^{b_{i}}},i=1,2$$

where *a_i_*, *b_i_*, and *c_i_* are the premise parameters.

Layer 2: Every adaptive node in this layer multiplies the incoming signal and sends the product out; the output is determined by:

(10) $$O_{2,i}=w_{i}=\mu_{A_{i}}\left(x\right)\mu_{B_{i}}\left(y\right),i=1,2$$

Layer 3: Ratio of the rules for firing strength to the sum of all rule’s firing strengths is given as:

(11) $$O_{3,i}={\bar{w}}_{i}=\frac{w_{i}}{w_{1}+w_{2}},i=1,2$$

Layer 4: In this layer, every adaptive node is a square node with the function:

(12) $$O_{4,i}={\bar{w}}_{i}f_{i}={\bar{w}}_{i}\left(p_{i}x+q_{i}y+r_{i}\right),i=1,2$$

where *p_i_*, *q_i_*, *r_i_* are the design parameters.

Layer 5: Fixed node computes the overall output as the summation of all incoming signals; the output is as follows:

(13) $$O_{5,i}=\sum_{i}{\bar{w}}_{i}f_{i}=\frac{\sum w_{i}f_{i}}{\sum {\;}_{i}w_{i}},i=1,2$$

#### 2.2.4. Local Correlation Maximization-Complementary Superiority (LCMCS)

To address all three issues considered in this study, the LCMCS method is proposed; the main steps are as follows:

(1) Spectral transforms. Spectral transforms help reduce the influence of noise; therefore, each REF is transformed into FDR, log(1/R) and (log[1/R])′.
(2) LCM analysis. To maximize the use of TN response information and eliminate the interference of noisy data, OSP and OCC of the original and transformed spectrum are obtained by LCM de-noising method, which has significant correlativity with TN content.
(3) Complementary superiority. OSP and measured TN values are used in PLS regression analysis, and several principal components (five principal components in this study) are acquired. These principal components and the measured TN contents are then used in ANFIS analysis, and the LCMCS models are established.
(4) Model-verifying. Sample data are used for model calibration and verification. In this study, from the 280 samples in each treatment, 150 samples were used for model calibration and the remaining 130 samples were used for model verification. Then, the best model was selected as the final model using the LCMCS method.

By carefully applying spectral transforms to wavelet, correlation, PLS regression, and ANFIS analysis methods, the LCMCS method can effectively remove noise while preserving the detail information, taking full advantage of useful spectral information and eliminating the interference of noisy data, and the complementary superiority between PLS regression and ANFIS are realized.

#### 2.2.5. Model Evaluation Standard

In this study, 150 soil samples were used to construct all models (55, 50 and 45 soil samples from subsided land of Cangzhou, Renqiu and Fengfeng, respectively), In addition, in order to fully validate the prediction abilities of all models, 130 soil samples were used in verification (45, 45 and 40 soil samples from subsided land of Cangzhou, Renqiu and Fengfeng, respectively) (Table 3). The stability and accuracy of all the models were determined by *R*^2^, root mean square error of calibration (RMSEC) and mean relative error of calibration (MREC). The estimation results were evaluated by root mean square error of validation (RMSEV) and mean relative error of validation (MREV). A good model will have a high *R*^2^, low root mean square errors (RMSEC and RMSEV), and small mean relative errors (MREC and MREV).

**Table 3:** Descriptive statistics of the calibration/validation set.

Dataset	NS		EP
Calibration	150	55 C	*R*^2^, RMSEC, MREC
		50 R	
		45 F	
Validation	130	45 C	*R*^2^, RMSEV, MREV
		45 R	
		40 F	

NS, Number of samples; C, Cangzhou City; R, Renqiu City; F, Fengfeng District; EP, Evaluation parameters.

## 3. Results and Discussion

### 3.1. Interpretation of Soil Spectral Reflectance

**Figure 5:**
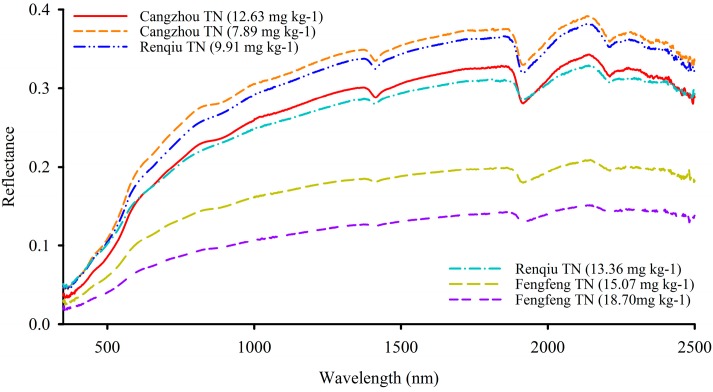
Original reflectance curve of soil samples with different TN contents.

Figure 5 shows the differences of spectral reflectance between spectra and samples with different TN contents (12.63, 7.89, 9.91, 13.36, 15.07 and 18.70 mg∙kg^−1^). The samples of the Fengfeng site had much more TN than samples of Cangzhou and Renqiu. Figure 5 also indicates that soil reflectance generally decreases with increasing TN content. A TN of 18.70 mg∙kg^−1^ shows lower reflectance values than the others, probably because of its greater TN content. In the entire visible-near-infrared spectrum, three remarkable water absorption peaks were observed at 1400, 1905 and 2200 nm. Although the differences of spectral characteristics caused by TN are apparent, it is still extremely difficult to reveal the relationships between spectra and TN content directly, especially when a greater number samples are considered. Organic nitrogen is a major constituent of SOM, therefore soil reflectance decreases possible correlation with SOM, which can affect estimation accuracy of TN prediction models obviously [21,80,81]. And the SOM interference would be left behind to further research. In this study, many processing algorithms were employed for the data mining and analysis.

### 3.2. OSP Acquisition

**Figure 6:**
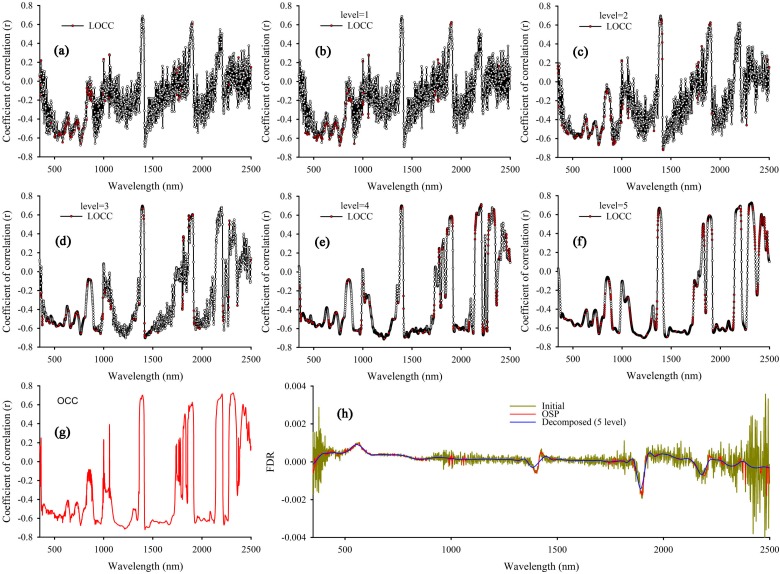
Wavelength dependence on coefficients of correlation between total soil nitrogen (TN) and first derivative differential of the soil spectra: initial (**a**); decomposed (1–5 levels) (**b**–**f**); optimal correlative curve (OCC) (**g**); and (**h**) first derivative differential reflectance curve of soil sample (Initial, decomposed [5 level] and the optimal spectra [OSP]).

Figure 6a shows the correlation coefficients between the measured TN content and the initial FDR (data of REF, log[1/R] and [log{1/R}]′ are not shown, the same as below), and the correlation coefficients of the measured TN content with decomposed FDR (1–5 levels; Figure 6b–f). Moreover, Table 4 gives maximum values of all the correlation coefficients of initial FDR and decomposed FDR. According to Figure 6a–f and Table 4, there is a stronger correlation when the level of wavelet decomposition is 5, whose maximum absolute correlation coefficient and average absolute correlation coefficient reach 0.725 (at 2316 nm) and 0.500. This implies that the wavelet analysis amplifies some useful TN information that is previously obscured by noise.

**Figure 7:**
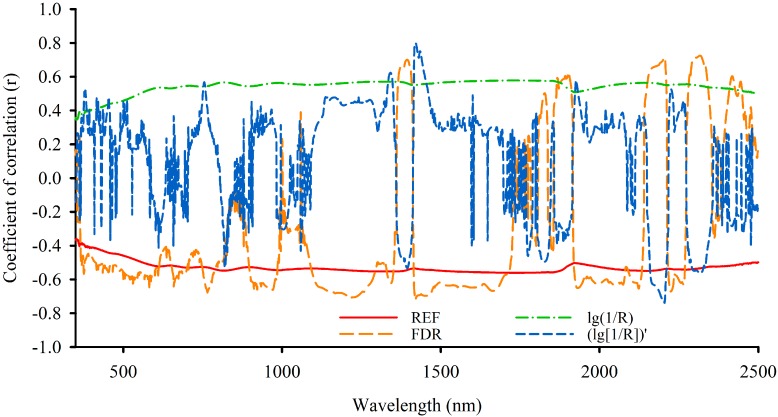
Optimal correlative curve of the original reflectance and its different transformation forms.

To preserve more detail during spectra de-noising, the optimal decomposition level of each band is found, which has the maximum correlation coefficient among the initial and decomposed spectra (1–5 levels) at each wavelength. The corresponding correlation coefficient and decomposed band are taken as LOCC and OB. The red points in Figure 6a–f show that the LOCC and the overall LOCC determine the OCC (Figure 6g). Figure 6h shows the initial FDR curve, decomposed FDR curve (5 level) and OSP, compared with initial FDR curve and decomposed curve (5 level). OSP can effectively remove noise while preserving the detail information simultaneously. Figure 7 shows all OCC of REF, FDR, log(1/R) and (log[1/R])′.

**Table 4:** Correlation analysis between total soil nitrogen (TN) and the first derivative differential FDR (initial and decomposed).

TSP	MPCB (nm)	CC	MNCB (nm)	CC	AACC
FDR	1397	0.669	766	−0.672	0.253
FDR (DL = 1)	1397	0.689	1419	−0.692	0.266
FDR (DL = 2)	1395	0.697	1421	−0.721	0.331
FDR (DL = 3)	1394	0.695	1422	−0.704	0.422
FDR (DL = 4)	2205	0.714	1214	−0.715	0.482
FDR (DL = 5)	2316	0.725	1223	−0.706	0.500

TSP, Types of spectral parameters; DL, Decomposition level; MPCB, Maximum positive correlation band; CC, Correlation coefficient; MNCB, Maximum negative correlation band; AACC, Average absolute correlation coefficient.

**Figure 8:**
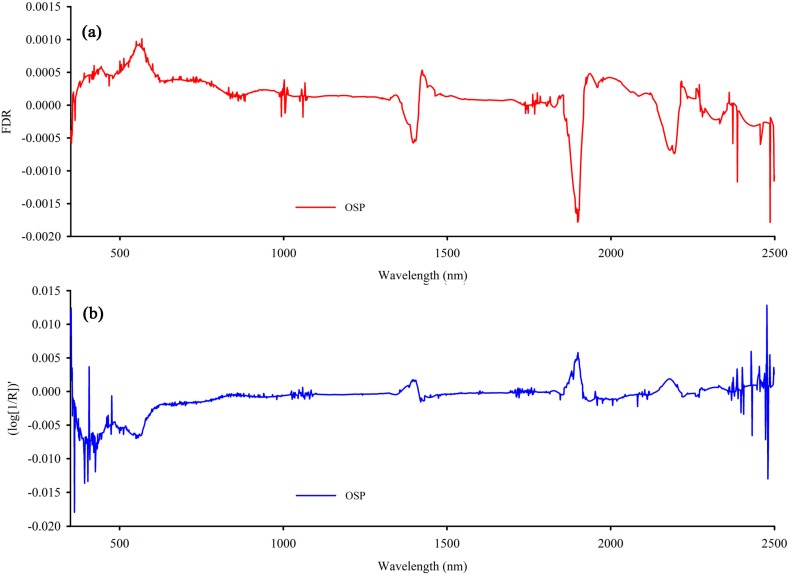
Optimal spectrum (OSP) of the first derivative differential (FDR) (**a**) and the first derivative differential of reciprocal logarithm (log[1/R])′ (**b**).

Based on Figure 7, the OCC of (log[1/R])′ performs better, and the correlation coefficient is 0.797. In addition, the OCC of FDR has more bands with high correlation than OCC of (log[1/R])′. Meanwhile, its maximum correlation coefficient is much higher than that of the OCC of REF and log(1/R). Table 5 gives their maximum correlation coefficients and number of bands at different levels of correlation. Therefore, OSP of FDR (Figure 8a and (log[1/R])′ (Figure 8b were used to build the LCMCS model.

As shown in Figure 8, the smoothness of spectral curves is obviously improved by LCM method, and spectral detail information is well preserved after de-noising, which indicates that the issue of how to reduce noise while retaining the details in hyperspectral data is solved satisfactorily.

**Table 5:** Comparisons of the optimal correlative curve (OCC) of the first derivative differential (FDR) and the first derivative differential of reciprocal logarithm (log[1/R])′.

TSP	CL	NB	MPCB (nm)	CC	MNCB (nm)	CC
FDR	**	2023	2316	0.725	1421	−0.721
	>0.40	1759	2316	0.725	1421	−0.721
	>0.45	1654	2316	0.725	1421	−0.721
	>0.50	1510	2316	0.725	1421	−0.721
	>0.55	1291	2316	0.725	1421	−0.721
	>0.60	949	2316	0.725	1421	−0.721
(log[1/R])′	**	1655	1422	0.797	2205	−0.739
	>0.40	566	1422	0.797	2205	−0.739
	>0.45	392	1422	0.797	2205	−0.739
	>0.50	210	1422	0.797	2205	−0.739
	>0.55	134	1422	0.797	2205	−0.739
	>0.60	92	1422	0.797	2205	−0.739

TSP, Types of spectral parameters; CL, Correlative levels; **, at the 0.01 significance level; NB, Number of bands; MPCB, Maximum positive correlation band; CC, Correlation coefficient; MNCB, Maximum negative correlation band.

### 3.3. Applicability of LCMCS Model

OSP and measured TN values were used in PLS regression analysis, and five principal components were acquired. These five principal components and the measured TN contents were then used in ANFIS analysis, and the LCMCS models were established. Table 6 shows a comparative analysis of the performance of various models established by the LCMCS method at different correlative levels of FDR (OSP) and (log[1/R])′ (OSP).

**Table 6:** Comparisons of the performance of models established by the local correlation maximization-complementary superiority method at different correlative levels of the first derivative differential (FDR (optimal spectrum [OSP]) and the first derivative differential of reciprocal logarithm (log[1/R])′ (OSP).

TSP	CL	LVs	Calibration (*n* = 150)			Validation (*n* = 130)		
			*R*^2^	RMSEC	MREC	*R*^2^	RMSEV	MREV
FDR	**	5	0.951	0.629	3.311	0.808	1.169	7.901
	>0.40	5	0.946	0.667	3.818	0.829	1.095	7.901
	>0.45	5	0.923	0.793	4.909	0.834	1.076	6.969
	>0.50	5	0.920	0.808	5.231	0.823	1.105	6.890
	>0.55	5	0.927	0.767	4.781	0.831	1.080	7.051
	>0.60	5	0.917	0.821	5.168	0.797	1.184	8.068
(log[1/R])′	**	5	0.991	0.269	1.446	0.885	0.898	5.921
	>0.40	5	0.939	0.704	4.220	0.681	1.529	9.613
	>0.45	5	0.910	0.854	5.009	0.817	1.123	7.602
	>0.50	5	0.953	0.616	3.615	0.785	1.240	8.178
	>0.55	5	0.954	0.608	3.037	0.779	1.234	7.626
	>0.60	5	0.957	0.588	2.968	0.776	1.255	7.815

TSP, Types of spectral parameters; CL, Correlative levels; **, at the 0.01 significance level; LVs, Number of latent variables.

Based on the 1655 selected effective bands of (log[1/R])′ (OSP), whose correlation coefficients were significant (*p* < 0.01), the optimal model of the LCMCS method was obtained and determined to be the final model of the LCMCS method, which produced more ideal results for both the calibration (*R*^2^ = 0.991, RMSEC = 0.269 and MREC = 1.446) and validation (*R*^2^ = 0.885, RMSEV = 0.898 and MREV = 5.921) analyses compared with other models. For the purpose of comparison, three issues were separately considered, and the corresponding solutions are as follows:

(1) PLS regression method. In PLS regression models, decomposed FDR (5 level) and (log[1/R])′ (4 level), whose correlation coefficients reached to 0.725 and 0.797, respectively, were used in PLS analysis. Based on the 1293 selected effective bands of (log[1/R])′ (5 level), whose correlation coefficients were significant (*p* < 0.01), the optimal model of PLS method was obtained, which was selected as the final model of the PLS regression method.
(2) Local correlation maximization method (LCM). Facing the second issue of how to reduce noise while retaining as much useful information as possible, OSP of FDR and (log[1/R])′ were used in PLS regression analysis. Based on the 1655 selected effective bands of (log[1/R])′ (OSP), whose correlation coefficients were significant (*p* < 0.01), the optimal model of the LCM method was obtained and selected as the final model of the LCM method.
(3) Complementary superiority method (CS). The CS model, which had the advantages of PLS regression and ANFIS, was aimed at addressing the third issue. The same PLS regression models, decomposed FDR (5 level) and (log[1/R])′ (4 level) were used. Based on the 382 selected effective bands of (log[1/R])′ (4 level), whose correlation coefficients were greater than 0.40, the optimal model of CS method was created and the final model of LCM method was determined.

Table 7 shows results of the best model found using each method.

**Table 7:** Test result of the local correlation maximization-complementary superiority method (LCMCS), complementary superiority (CS), local correlation maximization (LCM) and partial least squares regression (PLS) models provides for total soil nitrogen (TN) content.

Model	TSP	LVs	Calibration (*n* = 150)			Validation (*n* = 130/45 C/45 R/40 F)				
			*R*^2^	RMSEC	%MREC	*R*^2^	RMSEV		%MREV	
LCMCS	(log[1/R])′	5	0.991	0.269	1.446	0.885	0.898	0.861 C	5.921	6.463 C
								0.713 R		5.412 R
								1.103 F		5.883 F
LCM	(log[1/R])′	8	0.916	0.804	5.498	0.799	1.191	1.130 C	7.972	8.899 C
								0.863 R		6.839 R
								1.529 F		8.205 F
CS	(log[1/R])′	5	0.953	0.620	3.473	0.817	1.147	1.131 C	7.572	8.394 C
								0.945 R		6.958 R
								1.353 F		7.337 F
PLS	(log[1/R])′	8	0.830	1.141	7.756	0.747	1.373	1.354 C	9.525	10.38 C
								1.148 R		9.415 R
								1.608 F		8.683 F

TSP, Types of spectral parameters; LVs, Number of latent variables; C, Cangzhou City; R, Renqiu City; F, Fengfeng District.

**Figure 9:**
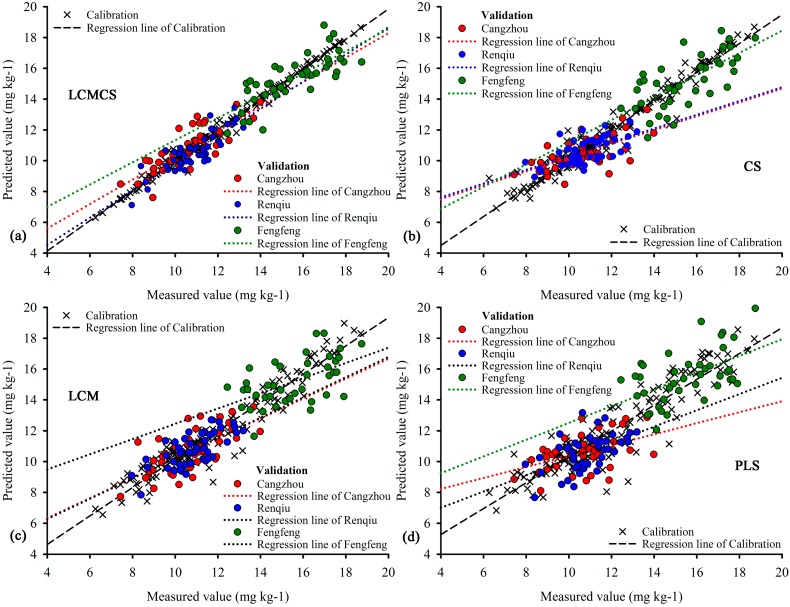
Comparisons of measured and predicted values by the local correlation maximization-complementary superiority method (LCMCS) (**a**); complementary superiority (CS) (**b**); local correlation maximization (LCM) (**c**) and partial least squares regression (PLS) (**d**) methods.

The PLS regression model provides good results in predicting TN contents (*R*^2^ = 0.747, RMSEV = 1.373, MREV = 9.525%; Table 7); this indicates that the PLS regression method based on spectral transforms and wavelet analysis is suitable for subsided land due to excessive extraction of different resources as discussed above. When the second issue was considered, the LCM model did perform better than the PLS regression model with the *R*^2^ of 0.799, RMSEV of 1.191 and the MREV of 7.972%; its accuracy to predict was obviously enhanced at all three sites, Changzhou, Renqiu and Fengfeng. Moreover, a small improvement occurred in the CS model when compared with the LCM model, although the precision in Renqiu was reduced from 6.839% to 6.958%. The results of the LCM and CS models indicate that when second and third issues were considered, the predictive effects can be improved significantly. However, it can be seen from the comparison that the LCMCS model (Figure 9a) produced lower prediction errors during both the calibration (*R*^2^ = 0.991, RMSEV = 0.269 and MREV = 1.446%) and validation (*R*^2^ = 0.885, RMSEV = 0.898, MREV = 5.921%) when compared with models built by other three methods (Figure 9b–d). Moreover, at all three sites, Cangzhou (RMSEV = 0.861, MREV = 6.463%), Renqiu (RMSEV = 0.713, MREV = 5.412%) and Fengfeng (RMSEV = 1.103, MREV = 5.883%), the estimation accuracy of the LCMCS model was also the closest to the ideal. In addition, overall models indicted that the estimation accuracy in Cangzhou was the poorest, followed by Fengfeng (except PLS model). The cause of this results and the influence degree of model estimation results from the land subsidence would be left behind to further research.

## 4. Conclusions/Outlook

By carefully applying spectral transforms as well as wavelet, correlation, PLS regression, and ANFIS analyses, the potential of the LCMCS method for the rapid quantification of TN was investigated. Based on the 1655 selected effective bands of (log[1/R])′ (OSP), whose correlation coefficients were significant (*p* < 0.01), the optimal model of the LCMCS method was developed as the final model of LCMCS method. For the purpose of comparison, three issues studied during model development.

The results show that all three methods compared could quantify TN efficiently. The LCM model and the CS model consider the second and third issue, respectively; their estimation results are more accurate than that of the PLS regression model. Between the LCM model and the CS model, the result of the CS model shows a small improvement. The LCMCS model, however, has the highest estimation accuracy because it considers all three issues together, which has been verified through all three study areas (Cangzhou, Renqiu or Fengfeng). In summary, the LCMCS method has great potential for use in monitoring TN in subsided lands due to excessive extraction of natural resources such as groundwater, oil and coal.
